# Immunopeptidomics in the cancer immunotherapy era

**DOI:** 10.37349/etat.2024.00249

**Published:** 2024-07-17

**Authors:** Sutatip Pongcharoen, Nongphanga Kaewsringam, Poorichaya Somaparn, Sittiruk Roytrakul, Yaowapa Maneerat, Komsak Pintha, Supachai Topanurak

**Affiliations:** Changchun Institute of Applied Chemistry, Chinese Academy of Sciences, China; ^1^Division of Immunology, Department of Medicine, Faculty of Medicine, Naresuan University, Phitsanulok 65000, Thailand; ^2^Department of Molecular Tropical Medicine and Genetics, Faculty of Tropical Medicine, Mahidol University, Bangkok 10400, Thailand; ^3^Center of Excellence in Systems Biology, Faculty of Medicine, Chulalongkorn University, Bangkok 10330, Thailand; ^4^Functional Proteomics Technology Laboratory, National Center for Genetic Engineering and Biotechnology, National Science and Technology Development Agency, Khlong Nueng, Khlong Luang 12120, Pathum Thani, Thailand; ^5^Department of Tropical Pathology, Faculty of Tropical Medicine, Mahidol University, Bangkok 10400, Thailand; ^6^Division of Biochemistry, School of Medical Sciences, University of Phayao, Phayao 56000, Thailand

**Keywords:** Immunopeptidomics, cancer immunotherapy, MHC ligandome

## Abstract

Cancer is the primary cause of death worldwide, and conventional treatments are painful, complicated, and have negative effects on healthy cells. However, cancer immunotherapy has emerged as a promising alternative. Principle of cancer immunotherapy is the re-activation of T-cell to combat the tumor that presents the peptide antigen on major histocompatibility complex (MHC). Those peptide antigens are identified with the set of omics technology, proteomics, genomics, and bioinformatics, which referred to immunopeptidomics. Indeed, immunopeptidomics can identify the neoantigens that are very useful for cancer immunotherapies. This review explored the use of immunopeptidomics for various immunotherapies, i.e., peptide-based vaccines, immune checkpoint inhibitors, oncolytic viruses, and chimeric antigen receptor T-cell. We also discussed how the diversity of neoantigens allows for the discovery of novel antigenic peptides while post-translationally modified peptides diversify the overall peptides binding to MHC or so-called MHC ligandome. The development of immunopeptidomics is keeping up-to-date and very active, particularly for clinical application. Immunopeptidomics is expected to be fast, accurate and reliable for the application for cancer immunotherapies.

## Introduction

Cancer is a devastating disease that causes millions of deaths worldwide. It is a complex condition with multiple factors contributing to its development. These factors include genetic mutations, environmental conditions, and viral infections [1–3]. The accumulation of genetic mutations and genomic instability are the main hallmarks of cancer, which can lead to a diverse range of malignant cell formations. This high genetic variability in cancer is referred to as tumor mutation burden (TMB). In the initial stages of cancer, the immune system can still recognize and eliminate malignant cells. However, as cancer progresses, tumor heterogeneity increases, causing the immune system to lose control, and cancer to advance to a more severe stage. Additionally, cancer immunosurveillance, a hallmark of cancer, plays a crucial role in promoting the survival and proliferation of malignant cells in advanced stages. Therefore, understanding the causes and hallmarks of cancer is crucial to developing effective treatments and improving patient outcomes.

When a tumor is formed, it creates a complex microenvironment known as the tumor microenvironment (TME), which acts as a protective barrier against the host immune system and promotes tumor survival [4]. Within this environment, the malignant cells of the tumor manipulate a specific type of immune cell called the regulatory T-cells (Treg) to prevent other immune cells from attacking the tumor. This process is called better understand how cancer interacts with the host immune system, the cancer immunity cycle provides a comprehensive view [5]. This cycle depicts the generation of anti-tumor and tumor-infiltrated T-cells within the TME to fight against malignant cells. The process begins with the dendritic cells capturing peptides released by the tumor and presenting them to immature T-cells. This process trains the T-cells to recognize cancerous cells. The activated cytotoxic T-cell ultimately eliminates the target tumors by recognizing the T-cell receptor (TCR) to the presented peptide by major histocompatibility complex (MHC). The complex of TCR-peptides and MHC triggers the secretion of T-cell cytokine to eradicate the target tumor, effectively halting the growth and spread of cancerous cells [6].

Cancer is a complex disease that arises from a variety of genetic mutations and alterations to the immune system. The body’s immune system is composed of both innate and adaptive pathways, and cancer can interfere with both of these pathways, leading to the generation of abnormal tumor-derived peptides from mutated proteins. These neoantigens, in turn, enable T-cells to distinguish between self and non-self antigens. MHCs are an important component of this process, as they help to present neoantigens to T-cells. TCRs selectively bind to different MHC allotypes in a process known as MHC restriction, with CD8^+^ T-cells recognizing MHC class I and CD4^+^ T-cells recognizing MHC class II. The binding and restriction of peptides to MHC are influenced by various physicochemical properties [7]. TMB, or tumor mutation burden, is a measure of the number of neoantigens present and the potential for T-cells to differentiate between self and non-self-antigens [8, 9]. However, cancer cells can also evade the immune system by suppressing the presentation of neoantigens through dysregulation of MHC expression in advanced stages of cancer.

Therefore, it is essential to restore the body’s ability to fight cancer cells through cancer treatment, such as immunotherapy. There are two main types of immunotherapies: adoptive T-cell transfer (ACT) and cancer vaccines [10, 11]. ACT uses cell-mediated immunity, while cancer vaccines use humoral immunity. The antigens presented by MHC are highly variable and depend on biological events inside the cancer cells. As a result, the T-cell antigen landscapes are dynamic, and they contribute to the expansion of T-cell repertoires. Understanding the correlation between the T-cell antigen landscape and cancer status can provide a deeper understanding of cancer immunotherapy. Genetic mutations and irregular gene expressions in malignant cells shape the neoantigen repertoire binding to MHC or peptide binding MHC (pMHC). The complete MHC binding peptides are collectively known as the MHC ligandome, and they play a crucial role in the presentation of neoantigens to T-cells [12, 13].

**Figure 1:**
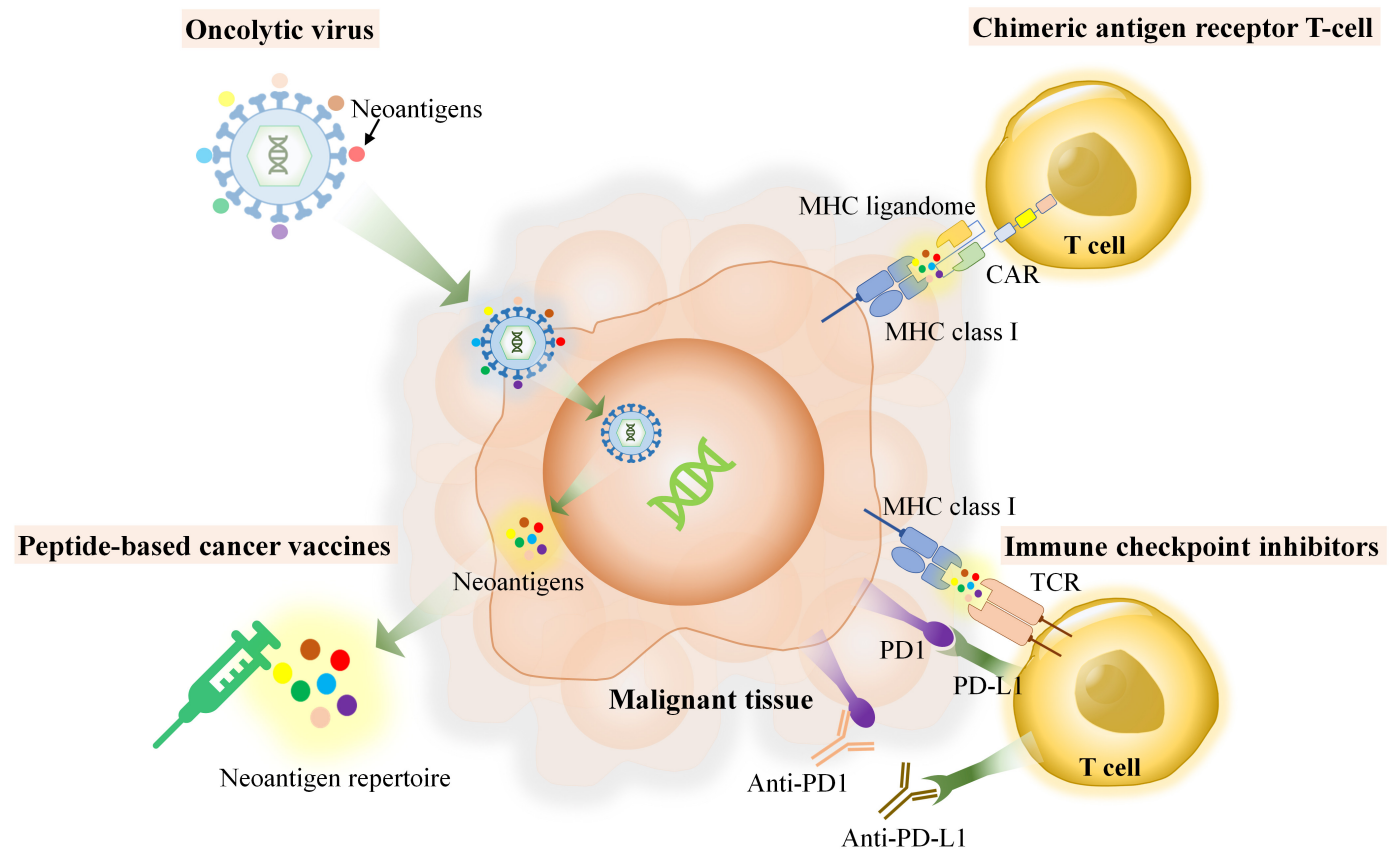
Role of immunopeptidomics or MHC ligandomes in various cancer immunotherapy platforms

T-cells play a crucial role in the immune defense of the body. They recognize specific antigens presented by MHC molecules. However, the diversity of T-cell antigens among and within tumors is influenced by the heterogeneity of these tumors. This implies that each individual has a unique collection of MHC-bound antigens, also known as the MHC ligandome. Despite this variability, there may still be some neoantigens that are shared among all individuals. The MHC ligandome serves as a mediator between innate and adaptive immunity, facilitating the recognition of tumor cells by immune cells. Neoantigen is self-antigen derived from tumor, herein, host immune can distinguish as foreign proteins, which have much dissimilarity of amino acid sequences and absent in the normal tissues. The source of neoantigens could be derived from unique proteins which arise though several machineries within host cell such as genomic mutation, in-del mutation, spliced variant mutation, gene fusion, non-coding region or unannotated open reading frame and RNA editing [14]. When proteins undergo non-synonymous coding region mutations, they may be degraded in the immunoproteasome, resulting in the production of degraded peptides. These peptides may then be transported to cancerous cells via MHC molecules. The discovery of various specific pMHCs from different MHC allotypes can be accomplished through immunopeptidomics, which is a combination of proteomics and bioinformatics used to study the MHC ligandome [15–17]. Immunopeptidomics is a rapidly growing field of research that focuses on identifying peptides that bind to MHC molecules and play a key role in immune responses. By studying the immunopeptidome or MHC ligand, researchers can gain insights into the complex interactions between cancer cells and the immune system. Although immunopeptidomics is not the recent technology, it has been emerging for more a decade, however, many of tools which use for immunopeptidomics require the improvements and further development, particularly, the accuracy and speed of the required technology such as next-generation sequencing (NGS), mass spectrometry (MS) and the essential bioinformatics for identification of MHC ligandome and neoantigens, in terms of personalized medicine. Application of immunopeptidomics relies use of neoantigens and neoantigen based-therapeutic application. As demonstrated in Figure 1, the applications of immunopeptidomics in a variety of immunotherapies, including peptide-based cancer vaccines, immune checkpoint inhibitors (ICIs), chimeric antigen receptor (CAR) T-cell therapy, and oncolytic viruses (OVs). This review explores the potential of immunopeptidomics to enhance cancer immunotherapy by providing a more comprehensive understanding of the MHC ligandome and its role in immune responses.

## Immunopeptidomics workflow

Cancer is characterized by the accumulation of genetic mutations, leading to the development of unique tumors for each patient. To break down mutated proteins resulting from somatic mutation, immunoproteasomes play a crucial role. Peptide fragments from protein degradation join MHC molecules and move to the malignant cell surface. TSAs, or tumor-specific antigens, differ from TAAs, or tumor-associated antigens, in that they come from the overexpressed proteins in malignant tissues. Neoantigens, on the other hand, are varied and derived from TSAs due to tumor heterogeneity [18]. Although genetic mutations are known to drive cancer progression, immune evasion mechanisms do not solely rely on these mutations. The recognition of mutated peptides that bind to MHC is necessary for T-cells, which means the expression of mutated genes into proteins is important for identifying neoantigens. Non-synonymous mutated coding regions can express corresponding mutated proteins that have the potential to be neoantigens, so detecting the expression of mutated genes is crucial for neoantigen discovery.

NGS is the primary method used to identify neoantigens. Mutated coding regions can be sequenced through whole exome sequencing (WES) and even mutated non-coding regions can be sequenced through whole-genome sequencing (WGS). Neoantigens can be canonical peptides derived from the mutation occurring in the coding region, or non-canonical peptides derived from the non-coding region mutation. Although MS and bioinformatics can identify the peptides from immunopurification, non-specific binding peptides from this method are relatively high. The binding of peptides to individual MHC allotypes properly attributes to induce T-cell activation against malignant tissues. MS and search engine identification can generate numerous peptides, but the selection of potential antigenic peptides is required for further identification of candidate peptides.

**Figure 2:**
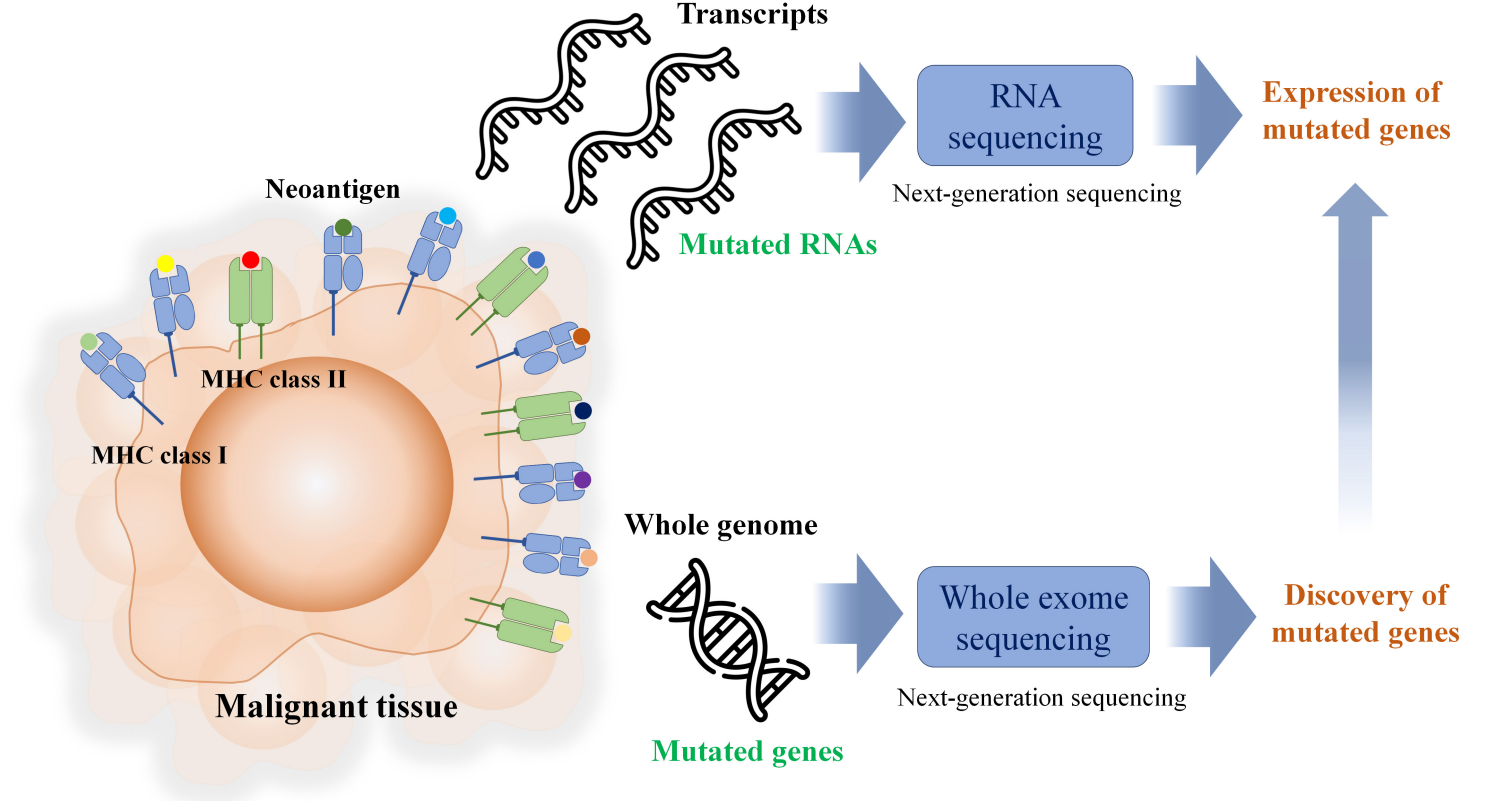
In brief neoantigen discovery workflow, next-generation sequencing is the key tool for identifying the somatic mutation and the expression of mutated genes including non-coding region

*In silico* MHC binding prediction is used for candidate selection, and Table 1 lists the freely available *in silico* MHC binding predictions. During the process of discovering neoantigens, RNA-seq is used to identify mutated coding genes, as depicted in Figure 2. However, it is crucial to note that RNA expression levels are not always indicative of protein expression levels. Furthermore, intracellular protein abundance is not a dependable predictor of the potential of pMHC or their immunogenicity. As a result, the current workflow for neoantigen discovery may not accurately predict the immunogenicity of pMHC.

**Table 1:** The example of bioinformatic tools for prediction of MHC binding peptides used in immunopeptidomics workflow

**Tools**	**Prediction**
NetMHCPan 4.1	Binding of peptides to any MHC class I with known sequence
NetMHCIIpan - 4.1	Binding of peptides to any MHC class II with known sequence
NetMHCphosPan - 1.0	Binding of phosphorylated peptides to any MHC with known sequence
NNAlign - 2.1	Generating artificial neural network models of receptor-ligand interactions
MHCMotifDecon - 1.0	Supervising method for motif deconvolution of MHC peptidome data
GibbsCluster	Server for unsupervised alignment and clustering of peptide sequences
MHCcluster - 2.0	Clustering MHC class I molecules (MHCI) based on their predicted binding specificity

**Figure 3:**
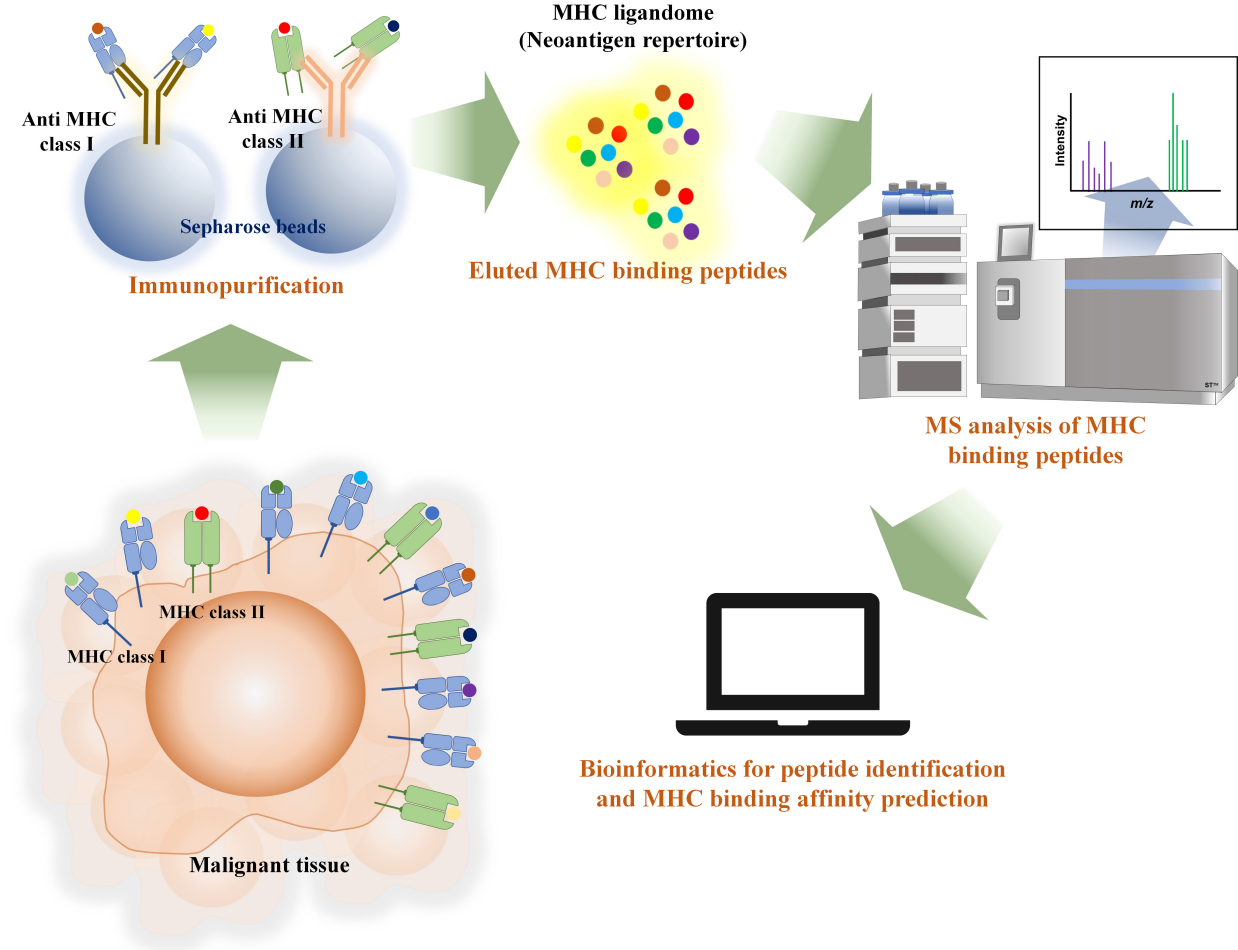
Immunopeptidomics workflow illustrated the extraction of MHC ligandome with immunopurification and mass spectrometry and bioinformatics are the tools for identifying MHC ligandome from malignant tissue and prediction of MHC binding affinity

On the other hand, the workflow for immunopeptidomics involves the extraction of pMHC through immunopurification or mild acid elution, as shown in Figure 3. This method aids in discovering the actual binding MHC peptide (pMHC) instead of relying solely on *in silico* prediction. However, the identification of eluted pMHC is only possible through MS-based proteomics. Therefore, accurate sequencing of the identified peptides is crucial for achieving better MHC binding prediction. Consequently, this process requires the use of a high-performance MS instrument.

Proteomic database search engines are essential tools for identifying peptides in cancer cells. These databases use algorithms to efficiently identify peptides, such as pMHC and mutated protein-derived peptides. Popular search engines like MSFragger, PEAKS, Proteome Discoverer, and Maxquant (Table 2) have developed these algorithms to quickly identify these peptides without requiring a specific proteolytic enzyme to cleave before MS analysis [19, 20]. PEAKS and MSFragger are especially useful as they can significantly increase the search speed.

**Table 2:** Database search engines and algorithms in database search engines used for identification of MHC binding peptides (pMHC) and post-translational modification (PTM) of identified peptides

**Search engines**	**DB search**	**De novo peptide sequencing**	PTM search^*^
Proteome Discoverer	SEQUEST	INFERY™	ProsightPD
Maxquant	Andromeda	MaxNovo	Andromeda
PEAKS	PEAKS	PEAKS BD	GlycanFinder
MSFragger	MSFragger	n/a	MSFragger-glyco
MetaMorpheus	MetaMorpheus	n/a	G-PTM-D
PepNoVo+	n/a	PepNOVO+	n/a
SMSnet	SMSnet	SMSnet	SMSnet

^*^ Database search for PTM can be phosphorylation, glycosylation, or other type of PTM

In regular proteomic approaches, MS results are searched against the reference protein database from the UniProt proteome databases. However, malignant cells often have variant and mutated proteins, making it essential to use personalized databases for searches. These databases are created using WES and RNA sequencing to identify variant proteins. Non-canonical peptides, which are referred to as neoantigens, originate from regions of the genome that do not code for proteins. The development of cancer and genetic mutations, as well as instability in the genome, can be attributed to frameshifts in open reading frames and a susceptibility to mutations in non-coding regions. Mutations in these regions lead to the production of non-canonical peptides that cannot be aligned with reference protein databases or customized ones. To investigate neoantigens derived from these non-canonical peptides, there are computational tools available that can generate peptide sequences independently, without relying on prior information from protein databases. Popular tools such as PEAKS and PepNovo+ commonly use de novo sequencing in the immunopeptidomics workflow [21]. Interestingly, there is an alternative tool called SMSnet that utilizes a deep learning-based hybrid de novo sequencing approach combined with database search. This tool enables the discovery of over 10,000 MHC binding peptides and human phosphopeptides [22].

**Figure 4:**
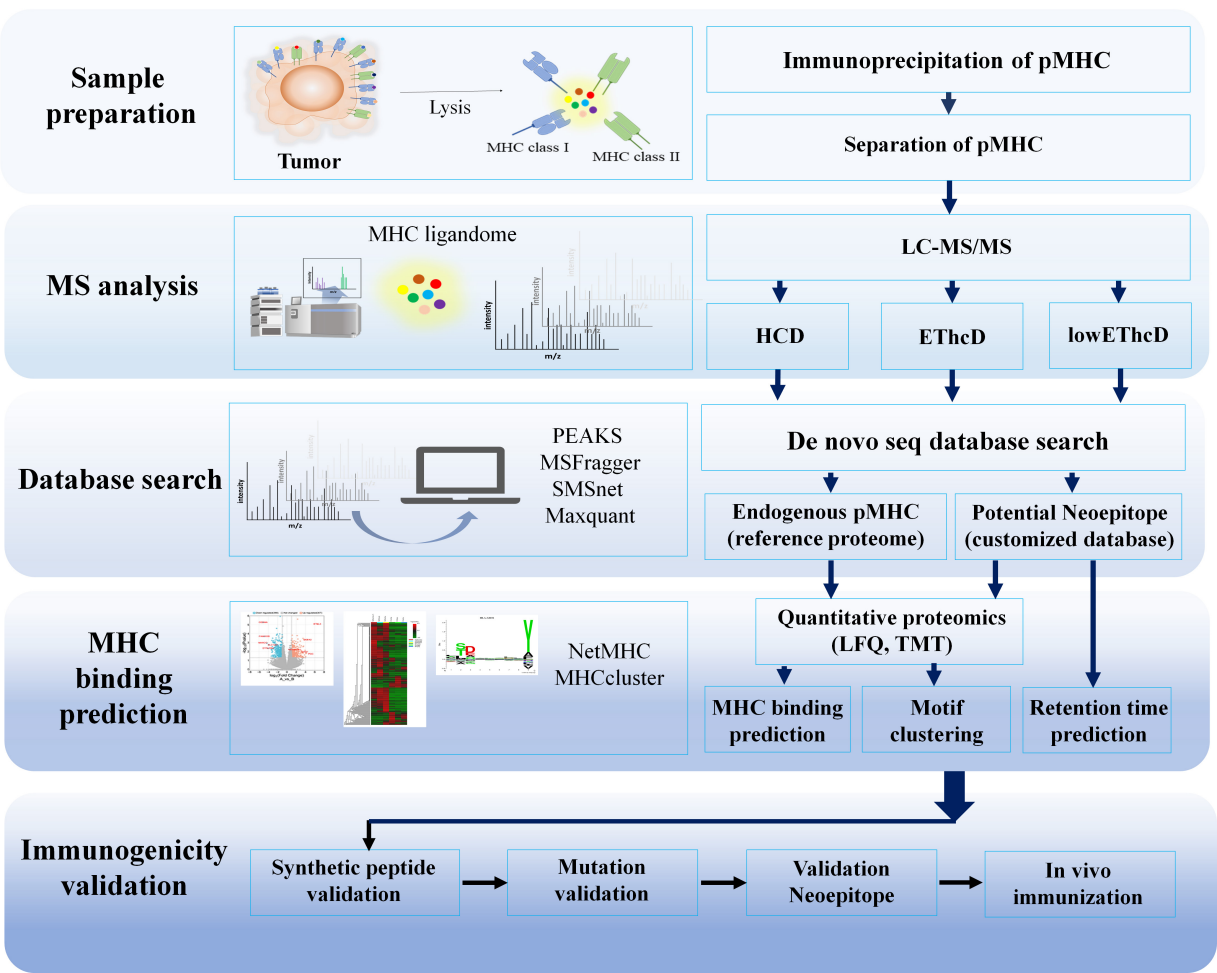
Immunopeptidomics workflow pipeline. HCD: high collision disassociation; EThcD: electron-transfer/higher-energy collision dissociation; LFQ: label free quantification; TMT: tandem mass tag

After identifying the peptides, it is crucial to determine which peptides can accurately bind to specific MHC allotypes. Computational analysis is used to predict the possibility of binding from the thousands of peptides discovered. *In silico* MHC binding and deconvolution, MHC binding techniques are used to identify immunogenic peptides before the immunogenicity test. The algorithms used for this purpose can be found in Table 1. Identifying immunogenic peptides is still a challenge due to the sheer number of peptides identified. However, advancements in cancer immunotherapy, MS instruments, bioinformatics tools, NGS like long-read Oxford nanopore, artificial intelligence, and deep machine learning algorithms, and the increase in training data to bind MHC algorithms show promise in improving and facilitating various cancer immunotherapy platforms, including clinical studies. The immunopeptidomics workflow pipeline was summarized in Figure 4.

## Application of immunopeptidomics for cancer immunotherapy platforms

### Immunopeptidomics for peptide-based cancer vaccines

Cancer vaccines are a promising approach to stimulate the immune system to recognize and kill cancer cells. Peptide-base cancer vaccine is the immunotherapy use peptide to activate the effector adaptive immunity and provide long-term gained immunity against a foreign tumor antigen peptide-base cancer vaccine is the immunotherapy use peptide to activate the effector adaptive immunity and provide long-term gained immunity against a foreign tumor antigen. Epitope-based cancer vaccines use epitope peptides to stimulate both humoral and cellular immune responses against tumor antigens. Epitope peptides are needed to help T-cells differentiate between self and non-self-antigens. Peptide-based cancer vaccines offer a more cost-effective and convenient manufacturing process compared to other cancer immunotherapies, with enhanced chemical stability. Proper binding with MHC is necessary for effective antigenic peptides to target malignancy cells. As previously stated, tumor heterogeneity leads to diverse immunogenic peptides and personalized treatment using peptide-based vaccines. Immunopeptidomics enables the identification of pMHC for personalized treatment. However, despite being the primary method for identifying neoepitope peptides from tumor biopsies, MS-based proteomics still has imperfect computational analysis for MS results. Because tumor genetics and antigen expression are unpredictable, using the same whole genome sequence as a database search reference could result in missing certain peptides. Additionally, post-translational modifications (PTMs) on the neoantigens, such as glycosylation, are likely overlooked due to their complex and diverse nature, even within the same glycosylation site. De novo sequencing helps identify neoepitope antigen peptides in non-canonical proteins, as well as the expression of non-coding regions and specific mutated peptides. The challenge of detecting neoantigens has led to a time-consuming and labor-intensive workflow, prompting the search for immunogenic peptides that can be used universally across all cancers and individuals, known as off-the-shelf peptides. Off-the-shelf antigenic peptides are needed for optimal T-cell activation when it comes to TSAs.

To date, for example, Provenge (Sipucleucel-T) is a peptide cancer vaccine that has been approved by FDA for patients with castration-resistant prostate cancer, with showing a partial tumor regression [23]. There are prophylactic cancer vaccines have been approved by the FDA, which are effective in reducing the burden of cancer-associated HPV and HBV, they cannot treat virus-infected tumor cells [24].

Previous study, in the case of HPV associated cancer and use of immunopeptidomics for further investigate viral oncoproteins binding to MHC allotypes, the researchers have developed a therapeutic cancer vaccine that can stimulate the immune system to target tumors or cancer cells that have been infected with a virus. These vaccines can target specific viral oncoproteins, such as HPV E6 and HPV E7, during infection to help treat tumors. Immunopeptidomics is a powerful tool used by scientists to identify and design individualized peptides that can more effectively target specific tumor tissues. Although HPV E6 and E7 were used as viral antigens for immunotherapy, HPV E1 has been identified as a potential peptide-based vaccine that can help fight against cancer-causing HPV. HPV can cause cancer in various organs, including oropharyngeal squamous cell carcinomas. Immunopeptidomics have identified other TSAs beyond the viral oncoprotein E6/E7 for the investigation of MHC ligandomes. These peptides can be stored in a warehouse for cancer vaccine design against oropharyngeal squamous cell carcinomas, particularly those caused by HPV [25, 26]. Although cancer vaccines targeting neoantigens have shown promising results and improved patient survival in recent years, personalized neoantigens are expensive and have limited application. Therefore, standard antigen discovery is an interesting topic for immunopeptidomics. Different tumor types and individuals may have varying limitations for the application of neoantigen-derived somatic mutation cancer vaccines. Peptide-based vaccines, specifically those derived from neoantigens, need to exhibit strong binding to HLA, high heterogeneity compared to wild type, expression in most tumor cells, and generation through mutations that impact survival. Despite these challenges, peptide-based cancer vaccines have more advantages than other cancer immunotherapies. However, they are less effective than simple peptides and require an adjuvant to boost immunity.

### Immunopeptidomics and ICIs

T-cells are a type of white blood cell that has the remarkable ability to differentiate between self and non-self by recognizing antigen peptides that are presented on MHC molecules. The immune system has developed a system of immune checkpoints that helps to distinguish between self and non-self to prevent any damage to healthy cells [27]. However, malignant cells can utilize immune checkpoints like PD1 (programmed cell death 1) to evade detection by T-cells. To combat this, monoclonal antibodies called ICIs have been designed to prevent the PD1 protein from binding to PD-L1 (programmed cell death ligand 1), which is a checkpoint protein found on T-cells. This enhances their ability to combat cancer cells.

The effectiveness of these inhibitors is dependent on the identification of non-self antigens. TMB refers to the number of mutations that are present in a tumor. A higher TMB indicates a greater number of neoantigens, which can assist T-cells in distinguishing between normal and malignant cells [28]. The efficacy of ICIs also depends on the genetic diversity of MHC allotypes, which are highly polymorphic [29]. These allotypes are responsible for presenting antigens to T-cells and the effectiveness of the inhibitors is determined by individual MHC ligandomes. Despite the increase in research on MHC ligandomes from clinical specimens, there is still a lack of clinical research on individual ICI treatment outcomes that rely on pMHC repertoire. It is important to note that ICIs are expensive and have a chance of failure. Biomarkers that indicate successful outcomes can be beneficial [30]. In addition to the quantity of checkpoint protein expression, neoantigens represented by TMB can be used as a predictive biomarker to measure the response to ICIs. The immunopeptidome landscape, which refers to all the peptides that can be presented by MHC molecules, could be a better indicator of ICI response than TMB alone. Indeed, there is a possibility of observing MHC deficiency in malignant tumors. Several studies have indicated that the downregulation of MHC class I in certain types of cancers is associated with resistance to ICIs and adoptive T-cell therapy [31–34]. Immunopeptidomics may not directly serve as predictive markers, but it might identify target antigens for CAR T-cell therapy, particularly for intracellular antigens. However, there has been no prior research using immunopeptidomics to develop predictive biomarkers. Previous studies have shown that the immunopeptidomics platform can aid in making informed decisions regarding immunotherapy for cancer patients, including personalized target selection [35]. In this study, the aim was to compare RNA expression and the copy number of HLA type binding peptides derived from immunopeptidomics data stored in a warehouse. They used qPCR measurement as a diagnostic tool for immunotherapy treatment. From a certain perspective, the identification of neoantigen peptides and the examination of innate immune system-related gene expression, along with a personalized analysis of MHC allotype genetics extracted from malignant tissues, have the potential to be effective predictors of immunotherapy efficacy.

### Oncolytic viruses (OVs) and immunopeptidomics

OVs are a new and exciting development in the field of cancer treatment [36]. They have the unique ability to select target cancerous cells or tumors while leaving healthy cells unharmed. Moreover, they can trigger metabolic changes in cancer cells that activate the immune system against cancer [37]. This is possible because OVs take advantage of the cancer-specific gene expression to cause the cell to rupture, releasing tumor-derived antigens that enhance the immune system’s ability to recognize and fight cancer. OVs are showing great promise as immunotherapy, especially when used in combination with other treatments [38, 39]. Solid tumors are notoriously difficult to treat with immunotherapy because the TME protects the cancer cells from the immune system. However, OVs are effective against solid tumors by infecting and reproducing within cancer cells, resulting in their destruction. The damaged cells release pathogen-associated molecular patterns (PAMPs) and damage-associated molecular patterns (DAMPs), including TAAs, which activate dendritic cells and stimulate T-cell responses against the infected tumor cells. The immunopeptidome plays a significant role in this process by identifying antigenic peptides that can activate CD8^+^ cytotoxic T-cells [39–42]. To improve the immunogenicity of peptide-based cancer vaccines, Peptide-coated Conditionally Replicating Adenovirus (PeptiCARD) technology has been developed, which displays immunogenic peptides on viral vector capsids [43]. However, using neoantigens has limitations since tumors have unique mutations, meaning multiple neo-epitope peptides are required to target them all. Current technology cannot deliver multiple peptides, so personalized OVs have been developed to overcome these limitations for cancer immunotherapy. The promising field of immunopeptidomics may help to optimize cancer immunotherapy further.

### Chimeric antigen receptor T cell (CAR-T cell)

The primary aim of CAR T-cell therapy is to overcome the challenge posed by the down-regulation of MHC in cancer immunosurveillance [44–46]. To do this, researchers genetically modify a patient’s cytotoxic T-cells using viral vectors, such as lentivirus or adeno-associated virus (AAV), to alter the expression of T-cells and co-immunostimulatory molecules [47–49]. These modifications include intracellular protein signaling in the T-cells. While TCR can recognize tumor antigens without relying on MHC antigen presentation, only a small fraction of the proteome contains membrane proteins. Some researchers express a limited number of membrane proteins on tumor cells, but mutated membrane proteins are rarely expressed on the tumor cell surface [50–53]. This presents a challenge for designing CARs, which currently rely on known glycoprotein surface antigens [54]. Patients who do not express these known protein antigens could face further challenges. Intracellular mutated proteins are a possible solution to facilitate T-cell visualization against tumor cells. However, the immunoproteasome degrades aberrant mutant proteins, providing a snapshot of those proteins located on the cell surface with MHC. Researchers can genetically engineer TCR to recognize tumor-specific aberrant proteins. Immunopeptidomics plays an essential role in visualizing antigen peptides that bind to MHC from intracellular proteins. Previous studies have shown that engineering TCR against immunogenic peptides that present MHC from patients’ tumors allowed for successful experiments. In the study conducted by Yarmarkovich et al. [51], the focus was on designing CAR-T cells for the treatment of neuroblastoma, a cancer that mostly affects young children [55, 56]. However, this type of cancer poses a significant challenge, as the tissue derived from neuroblastoma, which is developed from the nervous system, is often life-threatening. Additionally, neuroblastoma tumors have a low mutational burden and low MHC expression, making it difficult to treat with immunotherapies [57].

To overcome this challenge, the researchers employed immunopeptidomics, a technique that allowed them to discover and identify TSAs from eight neuroblastoma cell-derived xenograft (CDX) and patient-derived xenograft (PDX) models, showing a wide range of MHC expression. By using this technique, the researchers were able to identify approximately 7,000-8,000 peptides, which were then filtered down to 33 unique peptides derived from 29 unique proteins with a strong HLA-binding affinity [58].

The researchers chose specific antigens based on the expression of the protein in the tumor and its absence in normal tissues. They discovered that the peptide sequence QYNPIRTTF, derived from PHOX2B, could be used as an antigen for designing a peptide-centric CAR. During fetal development, PHOX2B expresses and becomes completely silenced in normal tissues before birth. The researchers found that this peptide was also found in HLA-A*23:1 and the highly divergent HLA-B*14:02. The study showed that peptide-centric CARs have a promising potential to treat intracellular oncoproteins and can be effective in treating cancers where MHC-restricted therapies have not been very successful. The use of immunopeptidomics has the potential to further improve other cancer immunotherapies.

All cancer immunotherapies have drawbacks that are restricted by many factors, such as host genetics, MHC polymorphism, the abundance of tumor-infiltrated T cells, MHC deficiency, low tumor mutation burden, low abundance of neoantigens or TAA, etc. Immunopeptidomics will be the means to alleviate the effectiveness of immunotherapies through investigation of MHC ligandome landscape. The drawbacks of immunotherapies that rely on immunopeptidome improvement are summarized in Table 3.

**Table 3:** Potential aids of immunopeptidomics to improve the efficacy of cancer immunotherapies

**Immunotherapy**	**Limitation and drawbacks**	**Potential immunopeptidomics aids**
Peptide-based cancer vaccines	The diversity of MHC genetics and binding of antigenic peptides must be specific, the binding, consequently, can elicit the T-cell response [73].	Design of synthetic peptides directly from patients [74].
Immune checkpoint inhibitors	Low neoantigen burden, low expression of immune checkpoint, and MHC deficiency may reduce the efficacy. Prediction of neoantigen uses only WES, which does not exactly correlate with pMHC [75].	Combination the treatment with personalized peptide-based vaccines or other cancer treatments and use of immunopeptidome for predictive biomarkers [76].
Oncolytic viruses	Difficulties in delivering the viral particles to the tumors, viral tropism targeting to the tumor, defense of cancer innate immune, OVs cannot sufficiently elicit T-cell response because of tumor heterogeneity [77, 78].	Identification of personalized pMHC and coat on OV capsid with personalized antigenic peptides for incline elicit T-cell response [41].
Chimeric T-cells (CAR T-cell)	CAR cannot recognize neoantigens derived intracellular mutated proteins [50].	Increase of CAR to visualize neoantigens intracellular proteins by discovery of personalized MHC ligandome [48].

WES: whole exome sequencing

## Immunopeptidomics and PTM

The formation of cancer is an intricate process driven by protein mutations, ultimately resulting in the emergence of neoantigens. Neoantigens are a protein subtype that arises as a result of somatic mutations. Nevertheless, it should be acknowledged that somatic mutation alone does not directly indicate abnormal PTM, which can also function as a neoantigen. Cancer cells can engage in PTMs, including phosphorylation, glycosylation, and ubiquitination. These modifications are acknowledged to have a profound effect on the cellular protein structure. The aforementioned alterations play a crucial role in distinguishing self-antigens from non-self-antigens for T-cells, enabling them to identify and combat cancer cells. Identifying abnormal PTM proteins is notably more formidable than detecting regular mutated proteins, creating a hindrance in effectively identifying cancer cells.

Genomic studies of tumors have revolutionized cancer research, providing us with a better understanding of tumor biology. While protein variants can be predicted directly from DNA sequences, PTMs cannot. Therefore, high-throughput MS is still the gold standard technique for analyzing PTMs and comparing them to DNA sequences. PTMs in malignancies differ from those found in normal tissues. The study of pan-cancer and database creation offers valuable data for cancer research in immunopeptidomics, which can help in the development of new and effective cancer treatments.

Geffen et al. conducted a comprehensive analysis of proteogenomics data from 1,100 patients across 11 cancer types, to investigate PTMs [59–61]. They collected a large dataset that revealed various PTM profiles related to cancer development, which are currently stored in the Clinical Proteomic Tumor Analysis Consortium (CPAC) database [61]. Studies have explored the relationship between PTMs and PTM-pMHC, where PTMs increase the immunogenicity of MHC ligands. Glycosylated and phosphorylated peptides are predominantly studied in PTM-pMHC research, indicating their significance in PTM dysregulation [62]. Glycoproteins are important components of the human proteome, and glycan structures play a vital role in biological processes such as cell communication, microorganism interactions, and immunity. Anomalies in glycosylation profiles can impact various diseases, including cancer. Preclinical research has observed a rise in O-linked glycosylation in neoplasms like cervical cancer, which is associated with viral infections [63]. MHC molecules are glycoproteins, and glycans on MHC molecules can strongly interact with antigens. Detecting glycosylated pMHC is still complex because peptide identification depends on a non-enzymatic search using a reference protein database, and only a mass shift can suggest potential glycosylation sites. Analysis can interrogate the glycosylated sites with deamidation at asparagine residue sites, in which the earlier study attempted to investigate formerly glycosylated proteins that have undergone deglycosylation via the ER-associated protein degradation (ERAD) pathway.

The computational method called HLA-glycol embedded in MSFragger to identify possible glycosylated pMHC from MS raw results [64]. This algorithm allows large-scale analysis of glycosylated pMHC in cancer with the improvement of search speed. This study may contribute to understanding the role of pMHC and its relationship to tumor biology. They can exploit comprehensive knowledge of the personalized design of the therapy against tumors. Glycosylation and phosphorylation in malignant cells increase the diversity of pMHC.

Phosphorylation is more reversible than glycosylation, especially in cancer cells. The study of phosphorylated peptides uses a standard enrichment method, but studying glycosylated pMHC is more difficult. In a previous study, researchers discovered phosphorylated pMHC from multi-MHC allotypes [65]. The prediction of pMHC binding affinity may need further improvements because the current pMHC binding prediction algorithm requires in-depth data training. Not only can PTMs increase the diversity of MHC ligandome, but they can also reshape MHC ligandome landscapes.

An earlier study employed a bioinformatic prediction tool to analyze PTM on MHC ligandome from published MHC ligandome data [66–68]. Due to the current approaches for neoantigen discovery or immunopeptidomics itself, particularly MS search engines for peptide identification, few of them can indicate types and sites of PTMs. MetaMorpheus and PEAKS PTM are examples of implemented search engines [69, 70]. This study suggests that PTM MHC ligandome can highlight the modified antigens to offer new therapeutic opportunities. Due to their instability, PTM detection is challenging, so it is crucial to consider cell sources, instruments, and lab settings before making it a routine analysis.

## The future direction of immunopeptidomics for cancer immunotherapy

Indeed, immunopeptidomics is not a new technology. It encompasses genomics, proteomics, and bioinformatics to identify antigenic peptides or neoantigen peptides. Each advancement in omics technology contributes to the enhancement of immunopeptidomics. While cancer immunotherapies are likely to underly on neoantigen discovery so that the accuracy and speed of immunopeptidomics, which is the tool for neoepitope antigen identification is supposed to be the must. In genomics, NGS is the primary platform used. Previously, most studies employed the short-read illumina platform to identify genetic mutations and analyze RNA expression. However, recently, third-generation sequencing technologies like nanopore and PacBio have emerged. These platforms offer a new perspective on completing genome sequencing, including the expression of circRNA, ncRNA, and lncRNA, which are types of genetic expression. Furthermore, the utilization of the long-read sequencing platform can greatly aid in the construction of personalized proteomic databases, facilitating the discovery of non-canonical peptides. Immunopeptidomics heavily relies on MS as its primary tool. MS plays a crucial role in the identification of pMHC complexes, encompassing both canonical and non-canonical peptides. It is worth noting that the majority of antigenic peptides are more likely to fall into the category of non-canonical peptides. To effectively detect these non-canonical peptides, MS must possess robust capabilities, as the sequences of amino acids they comprise may not be present in reference proteomic databases. In order to identify these non-canonical peptides, de novo sequencing may be required, necessitating that MS generate sufficiently high energy levels. The current disassociation energy used to fragment peptides, such as electron-transfer/higher-energy collision dissociation (EThcD), allows for the preservation of labile PTMs, such as doubly phosphorylated peptides. This preservation enables an increased discovery of aberrant PTMs in neoepitope peptides. Data acquisition methods, including DDA (Data Dependent Acquisition) and DIA (Data Independent Acquisition), as well as de novo sequencing, provide greater opportunities to uncover “dark matter” in proteomics, which can also be applied to immunopeptidomics. “Dark matter” in proteomics refers to the identification of expressed peptides that cannot be identified by current proteomic database search engines. Examples include peptides derived from ncRNA and those with unknown aberrant PTMs, such as irregular glycosylation. Previous studies have attempted to integrate RNA-seq derived protein databases into immunopeptidomics workflows, a concept known as “immunopeptidogenomics” [71]. In a recent study, a deep-learning machine embedded in PEAKS online was employed to facilitate the identification of these “dark matter” peptides [72]. As MS instruments continue to be updated at a rapid pace, we can expect an increase in the detection of personalized antigenic peptides, thereby improving both sensitivity and accuracy.

Bioinformatics is a rapidly evolving tool in the field of immunopeptidomics. It not only includes database search engines for identifying immunopeptides but also encompasses MHC affinity binding prediction tools. Since MHC genetic variations differ among different ethnicities, it is not feasible to rely on a single tool to predict the immunogenicity of a large number of pMHC from individuals. However, artificial intelligence shows great promise in being able to exploit this prediction in the near future. In summary, the tools available for immunopeptidomics are fast and have a wide range of applications in clinical settings. These include the development of peptide cancer vaccines, ICIs, OVs, and CAR-T cell therapies. Implementing immunopeptidomics in these platforms can significantly reduce the time, effort, and cost associated with producing and identifying neoepitope peptides.

## Conclusions

In advanced stages, cancer cells can avoid the immune system through immune evasion, enabled by the accumulation of somatic mutations that cause heterogeneity in malignancy. The absence of MHC in cancer cells allows them to avoid being detected by T-cells, highlighting the significance of enhancing the patient’s immune system to fight against the disease. Cancer immunotherapy is now recognized as a crucial component of cancer treatment, producing remarkable outcomes. Immunopeptidomics is a valuable tool for identifying antigen peptides, specifically neoantigens, in personalized immunotherapy. To enhance neoantigen-centered cancer immunotherapy, methods like individualized peptide-based cancer vaccines, predictive biomarkers for ICIs, personalized OVs, personalized intracellular antigen-based CAR-T cells, and immunopeptidomics can identify patient-specific neoantigens and improve treatment effectiveness. Immunopeptidomics is currently undergoing research and development. The development of NGS, MS technologies, and bioinformatics aims to enhance the quantity and precision of neoepitope peptides, particularly in proteomics’ “dark matter”. The speed of the technologies is also concerned for the clinical application. The use of immunopeptidomics is anticipated to greatly enhance cancer treatment in the near future.

## Abbreviations

CAR: chimeric antigen receptor
EThcD: electron-transfer/higher-energy collision dissociation
ICIs: immune checkpoint inhibitors
MHC: major histocompatibility complex
MS: mass spectrometry
NGS: next-generation sequencing
OVs: oncolytic viruses
PD1: programmed cell death 1
PD-L1: programmed cell death ligand 1
pMHC: peptide binding major histocompatibility complex
PTM: post-translational modification
TAAs: tumor-associated antigens
TCR: T-cell receptor
TMB: tumor mutation burden
TME: tumor microenvironment
TSAs: tumor-specific antigens
WES: whole exome sequencing
WGS: whole-genome sequencing

## Declarations

### Copyright

© The Author(s) 2024.
